# Impacts of Geri-Fit^®^ on Health Outcomes in Older Adults: An Exploratory Study

**DOI:** 10.3390/healthcare14131913

**Published:** 2026-07-01

**Authors:** Allyson Mark, Wei-Chen Lee, Hani Serag, Namita Bhardwaj, Michael Goodman, Carlos Clark, Hanaa S. Sallam

**Affiliations:** 1 Department of Population Health and Health Disparities, School of Public and Population Health, University of Texas Medical Branch, Galveston, TX 77555, USA; agmark@utmb.edu (A.M.); migoodma@utmb.edu (M.G.); hssallam@utmb.edu (H.S.S.); 2 Department of Family Medicine, John Sealy School of Medicine, University of Texas Medical Branch, Galveston, TX 77555, USA; weilee@utmb.edu (W.-C.L.); nabhardw@utmb.edu (N.B.); 3 Department of Internal Medicine, John Sealy School of Medicine, University of Texas Medical Branch, Galveston, TX 77555, USA; carclark@utmb.edu; 4 Department of Orthopedic Surgery and Rehabilitation, John Sealy School of Medicine, University of Texas Medical Branch, Galveston, TX 77555, USA; 5 Hospital Administration, University of Texas Medical Branch, Galveston, TX 77555, USA; 6 Department of Human Physiology, Faculty of Medicine, Suez Canal University, Ismailia 41522, Egypt

**Keywords:** aging, cholesterol, Geri-Fit^®^, Hemoglobin A1c, older adults, progressive resistance training

## Abstract

**Background/Objectives**: The Geri-Fit^®^ program, recognized by the National Council on Aging, is known to improve strength in older adults, yet it lacks robust evidence for clinical outcomes. The current study was performed to assess the change in clinical outcomes in addition to patient-reported change in mobility and general well-being **Methods**: A total of 227 adults aged 60 and older were recruited from clinics and community sites across Galveston and Harris counties and participated in 45 min classes twice weekly for 12 weeks, led by trained Geri-Fit^®^ instructors. A mixed-methods approach includes pre- and post-collection of biometric measures of Hemoglobin A1c, total cholesterol, weight, and waist circumference. Participants also completed mid- and post-program surveys, reporting changes in health behaviors, psychosocial outcomes, and physical changes, and provided qualitative feedback. **Results**: 44% of participants lost weight, nearly half reduced their waist circumference, 43.5% improved their Hemoglobin A1c, and total cholesterol decreased significantly (from 167.77 to 155.04 mg/dL; *p* = 0.02). Self-reported outcomes indicated that almost 100% of participants showed improvement or maintenance in mobility, strength, physical activity, and well-being. **Conclusions**: These findings suggest that Geri-Fit^®^ is associated with favorable clinical outcomes and improved functional health, supporting its potential as a community-based intervention to enhance physical activity, improve self-management, or reduce the risk of chronic disease among older adults.

## 1. Introduction

The US population is rapidly aging, with older adults (aged 65+) increasing from 4.7% to 16.8% in the last two decades and projected to exceed 20.9% by 2050 [1,2]. This demographic shift is associated with higher healthcare utilization, as older adults accounted for 40% of healthcare spending in 2023 [3,4]. With the median age of Americans rising from 30 years in 1980 to 39 years in 2022, the number of chronic conditions has increased [5]. Over 90% of older adults had at least 1 or more chronic conditions, with 78.8% reporting multiple chronic conditions such as high blood pressure, high cholesterol, and arthritis [6].

Chronic conditions can compound with the natural processes of aging and affect older adults’ ability to manage their health. As adults age, muscle power and performance decline rapidly after age 65 for women and 70 for men [7]. When functional capabilities and physical performance are compromised, balance and fall-related issues can occur. Each year, approximately $50 billion is spent on medical costs associated with non-fatal fall injuries, including hospital or nursing home care, rehabilitation, medical equipment use, and other professional services [8]. These costs do not include the long-term effects of falls or mobility-related injuries, including disability, lost time from other duties, and reduced quality of life [8]. Regular physical activity can help maintain physical functioning and reduce the risk of balance and fall-related injuries.

As of 2022, only 13.9% of older adults met federal physical activity guidelines for leisure-time aerobic and muscle-strengthening activities, which recommend 150 min of moderate-intensity physical activity and at least 2 days of muscle-strengthening activity every week [9]. The decline in strength with age can be slowed by regular participation in exercise to increase muscle strength [7,10]. Additionally, physical activity is closely associated with lowering the risk of developing or worsening chronic diseases such as heart disease, stroke, diabetes, or osteoporosis [9]. While many senior fitness programs focus on aerobic activity through walking or dance-style classes, strength training or resistance training is effective at slowing the muscle atrophy associated with aging [10].

One program that implements progressive resistance strength training for older adults is Geri-Fit^®^. It is a tier III evidence-based health promotion and chronic disease self-management support program recognized by the Administration on Aging (AoA) and the Administration for Community Living (ACL), a division of the U.S. Department of Health and Human Services (HHS) [11]. It is frequently recommended nationally as a resource for physical activity and fall prevention in older adults [12,13,14]. Geri-Fit^®^ is designed for older adults using functional strength-training workouts to rebuild and maintain strength. It uses a combination of strengthening exercises, stretching and range-of-motion exercises, stability and balance training, gait exercises, and cardiovascular activity.

The Geri-Fit^®^ program was integrated into The University of Texas Medical Branch (UTMB)’s suite of Patient Education Programs and expanded to community settings, including public libraries and faith-based organizations. The study findings may provide insights into demonstrating the multidimensional benefits of physical activity for older adults through an academic-community partnership. While this program is considered a standard by the National Council on Aging (NCOA), the evidence supporting its effectiveness is limited, and its impact on clinical health outcomes has not been thoroughly investigated. The purpose of this study is to examine UTMB’s experience in the field of implementation science related to participant enrollment, retention, and centeredness and evaluate the impacts of Geri-Fit^®^ on health outcomes (e.g., glycemic and cholesterol levels).

## 2. Materials and Methods

### 2.1. Data Source

A total of 227 participants aged 60 and older were recruited from UTMB clinics, senior centers, community-based organizations, and libraries where classes were held. Classes were delivered at various locations around the Gulf Coast, from Galveston Island up to Seabrook, spanning both Galveston and Harris counties. Classes were led by senior center employees, and UTMB employees trained and certified as Geri-Fit^®^ coaches. Participants underwent Geri-Fit^®^ for 45 min, twice weekly, for 12 weeks. Participants used a sturdy chair, a resistance band, a light set of dumbbells, and water during the classes. The Geri-Fit^®^ classes consist of a warm-up, a stretch, and a series of strength-training, range-of-motion, stability and balance, and gait exercises for the back and shoulders, legs, chest, arms, and abdominals, followed by a cool-down stretch.

### 2.2. Measurements

Participants were surveyed at several points throughout the program using a mixed-methods approach. Point-of-care Hemoglobin A1C (A1C), total cholesterol (TC), weight, and waist circumference (WC) were measured at baseline and after program completion. Halfway through the program and after program completion, participants took a Geri-Fit^®^-specific survey, reporting changes in mobility, overall health, overall strength, balance, standing or walking unassisted, energy levels, physical activity levels, and general well-being. Participants also provided qualitative feedback through open-ended questions about their experience with the program (Appendix A–Table A1). Participant surveys were collected via the Research Electronic Data Capture (REDCap^®^ v 16.1.14) tool hosted at the University of Texas Medical Branch [15,16].

### 2.3. Quantitative Data Analysis

Descriptive statistics were computed to summarize individual characteristics, using means and standard deviations for continuous variables and frequencies (percentages) for categorical variables. Bivariate analyses using *t*-test were conducted to examine changes in continuous measures (e.g., weight), and chi-square tests were conducted to examine changes in categorical outcomes (e.g., improved balance) at mid- and end-of-program, respectively. Fisher’s exact test was applied when the cell was smaller than 5. The effect size was calculated to assess the magnitude of differences in biometric outcomes between pre- and post-class measurements. All analyses were conducted using STATA v18 (College Station, TX, USA), and any *p*-value less than 0.05 was considered statistically significant.

### 2.4. Analysis of Open-Ended Questions

Participants’ responses to the open-ended questions were organized and classified using Microsoft Excel. Data were also reviewed by AM and W-CL, and any disagreements between the two authors were discussed and resolved by a team meeting. Themes emerged when certain topics came up repeatedly. The frequency for each theme was then reported to reflect patterns of their satisfaction and suggestions for our program.

## 3. Results

**Figure 1:**
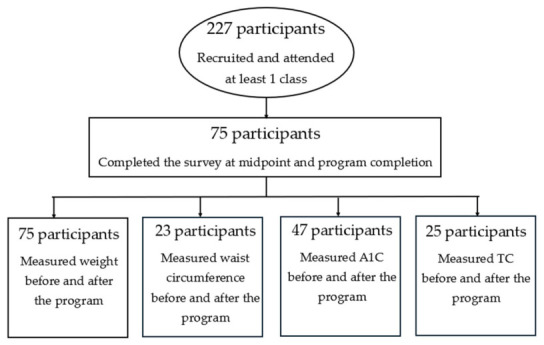
Flow Diagram of Participant Results.

All participants were presented with the same opportunity to complete the surveys and follow-up assessments across all program sites and instructors. However, of the 227 participants enrolled, 75 completed both surveys at the midpoint and after class completion (reported outcomes), as we honor participants’ right not to participate in data collection. Flow diagram of participant results is presented in Figure 1. Of the 75 participants, 90% were considered completers; however, some non-completers still showed improvements in their biometrics and reported outcomes. As suggested, we have acknowledged this limitation in our manuscript. Participants underwent assessments of weight, WC, A1C, and TC (measured outcomes). Participant characteristics are summarized in Table 1. The majority of participants were females, and the most common chronic condition was arthritis.

Table 2 provides measured changes in health outcomes, including weight loss, WC, A1C, and TC levels. Of the 75 participants who completed surveys, these variables were analyzed for those who completed the lab work using baseline and post-program values, excluding participants who missed lab appointments or declined to have lab values measured, such as A1C or TC. We observed that over 40% of participants decreased their A1C by an average of 0.9%, and 60% reduced their TC by an average of 7% (*p* = 0.02). The outcomes of TC and A1C had the largest effect size, indicating the validity of this biometric test.

Table 3 demonstrates the reported changes in participants’ physical strength and abilities. At each survey, participants were asked to evaluate their outcomes since starting Geri-Fit^®^. After program completion, all participants indicated improvement or maintenance in their strength, walking ability, physical activity, and driving skills. In addition, fewer people were afraid of falling, and more people reported improved well-being.

Table 4 summarizes participants’ reflections on this program. Around 59% (44/75) of participants reported maintenance or improvements in their physical function, including walking, standing, flexibility, and endurance. Also, 32% (24/75) indicated maintenance or improvements in their mental, behavioral, or social well-being, including feeling motivated and having fun. Three participants reported improvements in both domains. 40% of participants highlighted the program’s positive aspects, including great instruction and thoughtful coaches. Six participants offered suggestions for improvement, with mixed feedback; some preferred a longer program, while one suggested a shorter duration.

## 4. Discussion

This study examined the implementation and outcomes of the Geri-Fit^®^ program delivered through UTMB’s community partnerships across Galveston and Harris counties. It is the first to report statistically significant improvements in clinical outcomes with Geri-Fit^®^, namely, a 7% reduction in total cholesterol and a non-statistically significant 0.9% improvement in glycemic control, suggesting potential protective cardiometabolic effects of the program. Nearly all participants reported improvements or maintenance of strength, mobility, balance, and overall well-being, underscoring Geri-Fit^®^’s meaningful functional and psychosocial benefits for older adults in the community.

### 4.1. Measured Outcomes

We observed improvements in key measured outcomes, including TC, A1C, and WC, suggesting reduced cardiovascular risk and the potential prevention of further chronic diseases [17]. There was a significant 7% reduction in TC at the completion of Geri-Fit^®^, consistent with evidence that resistance training decreases TC in people with and without diabetes [18,19]. Resistance training enhances lipid metabolism by upregulating lipoprotein lipase activity. This increases the clearance of low-density lipoprotein from the blood, while simultaneously increasing muscle mass and resting metabolic rate, thereby facilitating more efficient fat processing [20].

A1C decreased by 0.9% at the completion of Geri-Fit^®^. Although not statistically significant, such a reduction suggests a clinically meaningful trend. Our findings are consistent with other reports indicating that resistance training reduces A1C levels in people with or without diabetes [21,22,23,24,25,26,27]. In fact, the reduction we observed was greater than that previously reported in people aged 60 and older with or without diabetes [28,29]. Resistance training is known to increase skeletal muscle mass [21], enhance glucose uptake via glucose transporter type 4, and improve insulin sensitivity [30,31].

Additionally, although the average WC increased and the result was not statistically significant, nearly half of the participants experienced a reduction in WC, a key marker of improved cardiometabolic risk [32]. Our findings were consistent with previous studies showing that strength training reduces WC by decreasing abdominal adiposity, including visceral fat accumulation around internal organs [33,34].

### 4.2. Reported Outcomes

The self-reported outcomes demonstrate consistently high rates of perceived improvement or maintenance across multiple domains of health and physical activity. All program completers reported improved or maintained ability to walk without assistance and to walk up and down stairs, reduced fear of falling, improved driving skills, increased level of physical activity, improved mobility, overall strength, and general well-being. Given the preservation and improvement of muscle strength and functional capacity to combat the aging processes, these findings align with the existing literature on the impact of resistance training in older adults [7,35,36,37]. We adopted the notion that in geriatric medicine and aging research, maintenance of function and improvement of function are often considered part of the same outcome domain—functional status or physical function—because preventing decline is itself a clinically meaningful benefit in older adults. The rationale is that in older adults, maintenance of physical function should be considered a clinically meaningful outcome alongside functional improvement. Given the expected age-related decline in mobility, strength, balance, and activities of daily living, interventions that preserve functional capacity may delay disability, support independent living, reduce healthcare utilization, and improve quality of life. Accordingly, the proposed study will evaluate the proportion of participants who maintain or improve physical function, recognizing both outcomes as indicators of intervention success. Maintaining function is particularly important for people with chronic diseases such as arthritis, diabetes, or heart disease, as it allows them to maintain or improve their physical activity capabilities to manage their health.

Importantly, the qualitative findings reinforced quantitative results. Our community-based Geri-Fit^®^ program is aligned with the framework of successful aging, increasing participants’ social engagement while cultivating healthy behaviors [38]. Participants described increased confidence, improved independence (e.g., navigating curbs and using public restrooms without assistance), enhanced flexibility, and better endurance. One-third of our participants stressed that the program structure enabled them to start exercise routines using feasible, evidence-based movements. Also, attending a group-based program made them have a sense of belonging, which greatly improved their motivation to stay in the program. These psychosocial benefits align with the literature, which finds that physical activity that provides social opportunities can enhance well-being, promote emotional health, and support successful aging [39]. Previous research on community-based programs has found that implementing physical activity or fitness programs in communities increases older adults’ psychological functioning and emotional connection with others, thereby improving quality of life [40,41]. These outcomes align with the multidimensional goals of a physical activity-based community program and highlight how the benefits to older adults extend beyond measurable biomarkers.

### 4.3. Feasibility

A key strength of this study is the real-world implementation of an evidence-based program through an academic medical center integrated with community partnerships. Developing Geri-Fit^®^ in libraries, churches, senior centers, and community organizations increased program accessibility and likely reduced barriers to participation. The UTMB implementation of the Geri-Fit^®^ program is consistent with other community-based exercise programs, demonstrating its accessibility and feasibility [42,43,44]. Training both UTMB staff and community partners ensures program fidelity between instructors and delivery sites while leveraging existing community infrastructure for older adults. Program evaluation is based on the Geri-Fit^®^ standardized tool so that the program outcome is comparable and reproducible to other institutions’. The program’s duration was effective in the community setting, with participants attending 14 of 24 sessions to be considered completers with a 90% completer rate, and participants attending an average of 18 sessions. The mixed methods design also strengthens the findings of this study. Objective biometric measures were complemented by patient-reported outcomes and qualitative feedback to provide a more comprehensive understanding of participants’ experiences and impact. Future work will expand this by interviewing administrators of community-based organizations to better understand how the Geri-Fit^®^ program benefits partner organizations and supports broader community impact.

### 4.4. Limitations

Limitations include high attrition and incomplete survey or laboratory follow-up. While 227 participants have matriculated through the Geri-Fit^®^ program, only 75 completed the surveys, and fewer still completed all laboratory follow-ups. While some of these data limitations can be addressed through improved follow-up, it is the participants’ right to decline to complete surveys or blood work, even after joining and completing the program. The participants who completed the surveys and measurements were consistent attenders, raising concern about potential selection bias. Participants who did not participate in data collection might have different outcomes, warranting caution when interpreting the findings from those who did not complete both surveys. In addition, the absence of a control group limits the ability to determine whether the observed changes are directly attributable to participation in Geri-Fit^®^. The population was also predominantly female (90.7%), which may limit the generalizability to older men. Moreover, self-reported measures may be subject to social desirability bias, particularly in this group-based intervention if strong instructor relationships are present. Changes in strength and mobility are not otherwise measured, so participant responses may be inconsistent with measurable outcomes. However, while some of these variables are self-reported and unverified, collecting self-reported data enables participants to reflect on and report their perceptions of the program and the changes they achieved.

## 5. Conclusions

This work highlights that the Geri-Fit^®^ model of structured resistance and functional strength training was associated with improved total cholesterol, favorable trends in glycemic control, and substantial self-reported gains in physical function, strength, and well-being among older adults. Beyond these outcomes, this program increases community awareness of the importance of regular exercise and strength training as essential components of healthy aging. As a community-based, physical activity intervention, Geri-Fit^®^ serves as a bridge between the health system and community to improve biometric outcomes, functional health, and chronic disease management. Its adaptable design allows Geri-Fit^®^ to align with diverse community cultures and partner effectively with a range of organizations, therefore promoting accessibility and supporting older adults aging in place. Overall, this implementation study provides applied, real-world data that scalable, evidence-based exercise programs may play a role in promoting functional independence by improving mobility and strength, enhancing perceived well-being and health, and potentially mitigating chronic disease risk and complications in this population. Further directions include expanding accessibility across geographic regions, strengthening integration with complementary services, such as nutrition education, and exploring sustainable funding pathways, including potential alignment with CMS reimbursement models.

## Figures and Tables

**Table 1:** Individual Characteristics of Geri-Fit^®^ Survey Participants (n = 75).

Characteristics	N (%) or Mean (Std.)
**Gender**	
Male	7 (9.3%)
Female	68 (90.7%)
Age (Range 60–89)	75.3 (0.74)
**Health Conditions**	
Hip or Knee Replacement	8 (10.7%)
Use a Cane, Walker, or Wheelchair	13 (20.0%)
Have Heart Diseases	16 (21.3%)
Have Diabetes	24 (32.0%)
Have Arthritis	50 (55.7%)

**Table 2:** Measured Changes in Health Outcomes Before and After Geri-Fit^®^.

Health Outcomes	Baseline	Post-Class	Change	Improved n/Total N (% People)	*p*-Value	Cohen’s d	Effect Size (r)
Weight (lbs) (n = 75)	177.14 (4.90)	176.18 (4.53)	−0.95 (1.44)	33/75 (44%)	0.8870	0.20	0.10
Waist Circumference (WC) (inches) (n = 23)	37.9 (1.21)	38.2 (1.26)	0.31 (0.53)	10/23 (44%)	0.5582	−0.24	−0.12
Hemoglobin A1c (A1C) (%) (n = 47)	6.33 (0.14)	6.24 (0.13)	−0.089 (0.10)	23/47 (49%)	0.3691	0.67	0.32
Total Cholesterol (TC) (mg/dL) (n = 25)	167.77 (7.22)	155.04 (6.49)	−11.72 (4.70)	15/25 (60%)	**0.0200**	1.85	0.68

**Table 3:** Changes in Physical Strength and Abilities. The number (N) and percentage (%) of people who reported “Improved” or “About the Same” in the survey.

	Mid-Point of Class N (%)	At Class Completion N (%)	*p*-Value
1. Geri-Fit^®^ Lifted Your Spirit or Put You in a Better Mood	71 (94.7%)	69 (92.0%)	0.513
2. Lost Weight	29 (38.7%)	29 (38.7%)	1.000
3. Better at Raising Arms Overhead	64 (85.3%)	65 (85.7%)	0.814
4. Mobility	74 (98.7%)	75 (100.0%)	1.000
5. Overall Health	72 (96.0%)	74 (98.7%)	0.620
6. Overall Strength	74 (98.7%)	75 (100.0%)	1.000
7. Balance	71 (94.7%)	74 (98.7%)	0.367
8. Stand Up without Assistance	74 (98.7%)	74 (98.7%)	1.000
9. Walk without Assistance	73 (97.3%)	75 (100.0%)	0.497
10. Walk Up and Down Stairs	73 (97.3%)	75 (100.0%)	0.497
11. General Well-Being	72 (96.0%)	75 (100.0%)	0.245
12. Energy Level	72 (96.0%)	74 (98.7%)	0.620
13. Level of Physical Activity	72 (96.0%)	75 (100.0%)	0.245
14. Fear of Falling Down	72 (96.0%)	75 (100.0%)	0.245
15. Changes in Pain	72 (96.0%)	73 (97.3%)	1.000
16. Changes in Memory	72 (96.0%)	74 (98.7%)	0.620
17. Driving Skill and Ability to Turn the Head	75 (100.0%)	75 (100.0%)	1.000
18. Hand and Finger Strength	74 (98.7%)	74 (98.7%)	1.000

**Table 4:** Feedback from Participants. The number (N) of participants indicating improvements responding to open-ended questions.

Subjects	N	Selected Quotes
Physical Improvement		I am able to step up and down a curb or stop without holding onto a railing or other support. I was not able to do this prior to Geri Fit. (ID-42)
	44	Stretch arms behind back easily now/Shoulders more flexible (ID-63)
		Geri-Fit^®^ has helped me maintain another activity (ID-75)
Mental, Behavioral, or Social Improvement		Geri-Fit^®^ has helped me continue walking miles a day. (ID-6)
	24	Exercise programs keep you to a schedule and keep you going. (ID-10)
		Have fun with other members like family. (ID-52)
Both Physical and Mental Improvements		Physically I feel better and have a more positive attitude towards aging. (ID-40)
	3	Better balance and a feeling of more confidence since I feel stronger (ID-41)
		Can use a public bathroom again without a handrail, improve self-confidence, assertiveness, speak up for myself when I need to, make friends easier. (ID-57)
Other Positive Aspects of Geri-Fit^®^		The coach did an awesome job of changing things and increasing reps as we went along. (ID-12)
	30	My coach is outstanding! She is so thorough, patient, & kind. She even helps us do the exercises correctly, so we get the most benefit from them. She is superb! Can this class please be offered again next semester? Thank you! (ID-55)
		Instruction has given me a whole new range of exercises to do at home. (ID-74)
		Keep it going. (ID-7)
Suggestions	6	Music could be used. (ID-24)
		Have weekly classes with no breaks. (ID-34)
		Need more exercise programs at senior center. (ID-51)
		Hope to do it year around. (ID-61)
		Would rather have 8 weeks instead of 12 weeks. (ID-68)

## Data Availability

The de-identified data is available upon request. The data are not publicly available as we are still working on a data platform for public availability of data.

## Abbreviations

The following abbreviations are used in this manuscript:

A1C	Hemoglobin A1c
CMS	Centers for Medicare & Medicaid Services
TC	Total Cholesterol
UTMB	University of Texas Medical Branch
WC	Waist Circumference

## Appendix A

**Table A1:** Geri-Fit^®^.

Demographic Information	
First Name	
Last Name	
Current Height (in inches)	
Current Weight (in lbs)	
Sex	
**Program Information**	
What date did you start attending Geri-Fit?	
How many sessions have you finished in Geri-Fit?	
In which city and state did you participate in Geri-Fit?	
Please tell us about your current condition(s).	Options: Yes, No
Have you had a hip or knee replacement in the past 5 years?	
Do you use a cane, walker, or wheelchair to get around?	
Do you have heart disease?	
Do you have diabetes?	
Do you have arthritis?	
Did Geri-Fit help lift your spirits or put you in a better mood?	
Did you lose any weight while enrolled in Geri-Fit?	
Are you able to raise your arms overhead better than when you first started in Geri-Fit?	
If you answered yes to any of the conditions above (heart disease, diabetes, arthritis), would you say that any of your health conditions have improved since starting Geri-Fit?	
If you answered “yes” to the question about weight loss, how many pounds did you lose?	
Since starting Geri-Fit, please rate the following:	Options: Improved, The Same, Gotten Worse, Not Applicable or Never had these conditions
How is your mobility?	
How would you rate your health overall?	
How would you rate your overall strength?	
How would you rate your balance?	
How would you rate your ability to stand up without assistance?	
How would you rate your ability to walk without assistance?	
How would you rate your ability to walk up and down the stairs?	
How is your general well-being?	
How is your energy level?	
How is your fear of falling down?	
Have you noticed any changes in your “pain” (such as pain from arthritis, back pain, stiffness in joints)?	
Have you noticed any change in your memory?	
Any changes in your driving skills and ability to turn your head around?	
Would you recommend the Geri-Fit program to your friends and family members?	Options: Yes, No
Final Thoughts and Comments, such as your personal experience.	Open-ended, free response question.
